# Microsurgical i-Trainer: a low cost method to replicate a microscope

**DOI:** 10.1308/003588413X13511609957056i

**Published:** 2013-01

**Authors:** Kavit Amin, Victoria Teoh, Barbara Jemec

**Affiliations:** Royal Free Hospital, Pond Street, London

Microsurgical training is fundamental to training in plastic and reconstructive surgery. Innovative methods are required that are simple, accessible and cheap to replicate intraoperative experience as well as enable practice in this technically demanding activity. Prior to embarking on loupes-only microsurgery, it has been suggested that intensive training under the microscope is essential.^1^ This simple method provides medical students and junior trainees without loupes to practise opera-tive skills before dedicating themselves to ownership of loupes.

**Figure 1:**
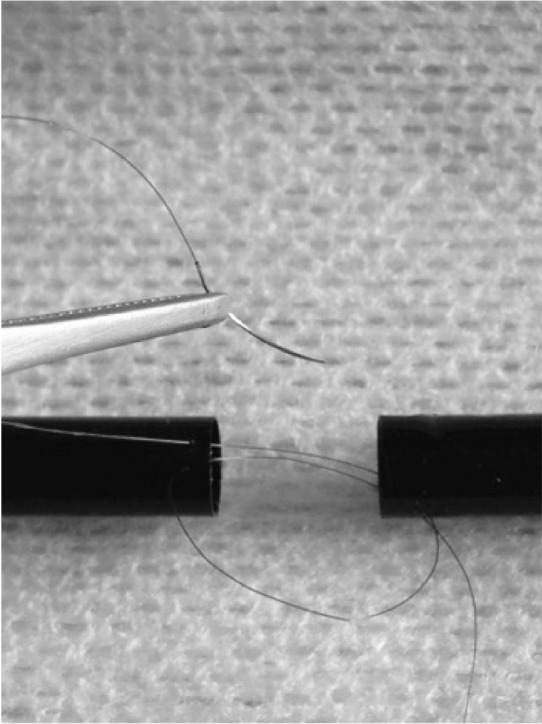
Using iPad^®^ at 3x zoom: anastomosing two ends of a fine straw with a 7/0 Prolene^®^ suture

We suggest downloading an application that uses the zoom function on Apple’s iPad^®^ (5 megapixel lens with autofocus), and mounting the iPad^®^ onto a wooden frame to replicate loupes and a microscope. Applications such as ‘Camera Zoom’ (available for free from iTunes^®^) allow up to 4x magnification (Fig 1). Operative footage can be recorded at the same time in high definition (1080p), consisting of up to 30 frames per second with audio. Footage can be reviewed later with senior colleagues as a teaching opportunity.
